# $F(\psi,\varphi)$-Contraction in terms of measure of noncompactness with application for nonlinear integral equations

**DOI:** 10.1186/s13660-017-1545-2

**Published:** 2017-10-30

**Authors:** Farzaneh Nikbakhtsarvestani, S Mansour Vaezpour, Mehdi Asadi

**Affiliations:** 1 Young Researchers and Elite club, Shiraz Branch, Islamic Azad University, Shiraz, Iran; 2 Department of Mathematics and Computer Sciences, Amirkabir University of Technology, Tehran, Iran; 3 Department of Mathematics, Zanjan Branch, Islamic Azad University, Zanjan, Iran

**Keywords:** 47H10, 34A12, 54H25, fixed point, measure of noncompactness, $F(\psi,\varphi)$-contraction

## Abstract

In this paper, some new generalization of Darbo’s fixed point theorem is proved by using a $F(\psi,\varphi)$-contraction in terms of a measure of noncompactness. Our result extends to obtaining a common fixed point for a pair of compatible mappings. The paper contains an application for nonlinear integral equations as well.

## Introduction and preliminaries

A contractive condition in terms of a measure of noncompactness, which was first used by Darbo, is one of the fruitful tools to obtain fixed point and common fixed point theorems. The extensions of these contractions in linear and integral type which are known as generalizations of Darbo’s fixed point theorem, are considered by many authors; see, for example, [1–9] and the references therein.

Recently, Khodabakhshi [6] obtained some new common fixed point results with the technique associated with a measure of noncompactness for two commuting operators.

Inspired by the class of *α*-*ψ* contractive type mappings which was introduced by Samet *et al.* [10], Ansari [11] presented the weaker class of this contraction named $F(\psi,\varphi)$-contraction and used it to obtain fixed point and common fixed point results.

This paper mainly aims at employing the $F(\psi,\varphi)$-contraction and its property in terms of a measure of noncompactness to investigate a fixed point and a common fixed point for a pair of compatible mappings.

Now we present some definitions, notations and results which will be needed later. Throughout this paper we assume that *E* is an infinite dimensional Banach space. If *C* is a subset of *E* then the symbols $\overline{\operatorname {co}} (C)$ and $\mathfrak{M}_{E}$ and $\mathfrak{N}_{E}$ denote the closure of convex hull of *C* and the family of nonempty bounded subsets of *E* and the subfamily consisting of all relatively compact subsets of *E*, respectively.

The measure of noncompactness was introduced by Kuratowski [12],

$$ \mu( S ) := \inf \Biggl\{ \delta> 0 : S =\bigcup_{i=1}^{n} S_{i} \text{ for some } S_{i} \text{ with } \operatorname {diam}( S_{i} ) \leq\delta \text{ for } 1 \leq i \leq n< \infty \Biggr\} , $$

for a bounded subset *S* of a metric space *X*.

From now on we will use the following definition for the measure of noncompactness.

### Definition 1.1

([13])

A mapping $\mu: \mathfrak {M}_{E} \to[ 0,\infty)$ is said to be a measure of noncompactness in *E* if it satisfies the following conditions:

1. $\emptyset\neq \operatorname {Ker}\mu= \{ X \in \mathfrak {M}_{E} : \mu( X ) = 0 \} \subseteq\mathfrak{N}_{E}$;
2. $X \subseteq Y\Rightarrow\mu( X ) \leq\mu( Y )$;
3. $\mu(\overline{X}) = \mu(\operatorname {co}X) =\mu( X )$;
4. $\mu(\lambda X + ( 1 -\lambda) Y ) \leq\lambda\mu( X ) + ( 1 -\lambda)\mu( Y )$ for $\lambda\in[ 0 , 1 ]$;
5. If $\{X_{n}\}$ is a sequence of closed sets from $\mathfrak {M}_{E}$ such that $X_{n + 1}\subseteq X_{n}$, ($n \geq1 $) and if $\lim_{n\to\infty} \mu( X_{n} ) = 0$ then the intersection set $X_{\infty}=\bigcap_{n=1}^{\infty}X_{n}$ is nonempty.

The family Ker*μ* described in (A1) is said to be the kernel of the measure of noncompactness *μ*. Observe that $X_{\infty}\in \operatorname {Ker}\mu$, since $\mu(X_{\infty}) \leq\mu(X _{n})$ for any *n*.

### Definition 1.2

([14])

A function $\psi:[0,\infty) \to[0,\infty)$ is called an altering distance function if the following properties are satisfied:

1. *ψ* is nondecreasing and continuous;
2. $\psi(t)=0$ if and only if $t=0$.

We denote by Ψ the class of altering distance functions.

### Definition 1.3

([11])

An ultra altering distance function is a continuous, nondecreasing mapping $\varphi:[0,\infty) \to[0,\infty)$ such that $\varphi(t)>0$ for $t>0$ and $\varphi(0)\geq0$.

We denote by Φ the class of ultra altering distance functions.

### Definition 1.4

([11])

A mapping $F:[0,\infty)^{2} \to\mathbb{R}$ is called a *C*-class function if it is continuous and satisfies the following axioms:

1. $F(s,t)\leq s$;
2. $F(s,t)=s$ implies that either $s=0$ or $t=0$; for all $s,t \in[0, \infty)$.

Note for some *F* we have $F(0,0)=0$.

We denote *C*-class functions by $\mathcal{C}$.

### Definition 1.5

([15])

A pair of self-mappings *F* and *G* on *X* is weakly compatible if there exists a point $x \in X$ such that $F(x)=G(x)$ implies $FGx=GFx$ i.e., they commute at their coincidence point.

### Proposition 1.6

([16, Proposition 1.5])

*Let* *f* *and* *g* *be weakly compatible self*-*mappings of a set* *X*. *If* *f* *and* *g* *have a unique point of coincidence*, $w=f(x)=g(x)$. *Then* *w* *is the unique common fixed point of* *f* *and g*.

### Lemma 1.7

([17])

*Let* *X* *be a nonempty set and* $f: X \to X$ *be a function*. *Then there exists a subset* $E \subseteq X$ *such that* $f(E)=f(X)$ *and* $f:E \to X$ *is one to one*.

Now, we mention the following two theorems stated in [13, 18].

### Theorem 1.8

(Schauder [18])

*Let* *C* *be a closed*, *convex subset of a Banach space* *E*. *Then every compact*, *continuous map* $F : C \to C$ *has at least one fixed point*.

As a significant generalization of Schauder’s fixed point theorem, we have the following fixed point theorem.

### Theorem 1.9

(Darbo [13])

*Let* *C* *be a nonempty*, *bounded*, *closed*, *and convex subset of a Banach space* *E* *and let* $T : C \to C$ *be a continuous mapping*. *Assume that there exists a constant* $k \in[ 0 , 1 )$ *such that*

$$ \mu \bigl( T ( X ) \bigr)\leq k \mu( X ) $$

*for any subset* *X* *of* *C*. *Then* *T* *has a fixed point*.

## Main results

This section starts by some of the theorems and corollaries related to fixed point are obtained by using $F(\psi,\varphi)$-contraction in terms of a measure of noncompactness. Next, for a pair of compatible mappings a common fixed point theorem is considered. In the sequel, theorems are proved in integral type to obtain a fixed point and a common fixed point. Our results generalized Darbo’s fixed point theorem and a fixed point theorem which was recently proved.

### Theorem 2.1

*Let* *C* *be a nonempty*, *bounded*, *closed*, *and convex subset of a Banach space* *E* *and let* $T: C \to C$ *be a continuous mapping*, *such that*

$$ \psi \bigl(\mu \bigl(T(M) \bigr) \bigr)\leq F \bigl(\psi \bigl(\mu(M) \bigr), \varphi \bigl(\mu(M) \bigr) \bigr), $$

*for any subset* *M* *of* *C* *and where* $\psi\in\Psi$, $\varphi\in \Phi$ *and* $F \in\mathcal{C}$. *Then* *T* *has a fixed point*.

### Proof

Define a sequence $\{C_{n}\}_{n=0}^{\infty}$ setting

$$ C_{0}:=C, \quad\quad C_{n}:=\overline{\operatorname {co}} T(C_{n-1}), $$

where $n=1,2,\ldots$ . Now let us prove that

1 $$ C_{n+1} \subseteq C_{n}, \quad\quad T(C_{n})\subseteq C_{n}, $$

for every $n=0,1,\ldots$ . The first inclusion will be proved via mathematical induction. Let $n=0$. Since $C_{0}=C$, *C* is convex and closed, $T(\cdot):C \to C$, we have $C_{1}=\overline{\operatorname {co}}(T(C_{0})) \subset C_{0}$. Now assume that $C_{n} \subset C_{n-1}$. Then $\overline{\operatorname {co}}(T(C_{n}))\subset\overline{\operatorname {co}}(T(C_{n-1}))$. So we obtain $C_{n+1} \subset C_{n}$. The second inclusion follows immediately from the first one, $T(C_{n})\subset\overline{\operatorname {co}}(T(C _{n}))=C_{n+1}\subset C_{n}$.

If there exists $N \in\mathbb{N}$ such that $\mu(N)=0$ then $C_{N}$ is compact and Schauder’s fixed point theorem ensures that *T* has a fixed point in $C_{N}$ where $C_{N} \subset C$. Suppose $\mu(C_{n})>0$ for each $n \in\mathbb{N}$.

Taking into consideration that $F \in\mathcal{C}$, the property (A3) from Definition (1.1) and using the inequality given in the theorem, we have

2 $$\begin{aligned} \begin{aligned}[b] \psi \bigl(\mu(C_{n+1}) \bigr) &=\psi \bigl(\mu \bigl( \overline{\operatorname {co}} \bigl(T(C_{n}) \bigr) \bigr) \bigr) \\ &=\psi\bigl(\mu \bigl(T(C_{n}) \bigr)\bigr) \\ &\leq F \bigl(\psi \bigl(\mu(C_{n}) \bigr),\varphi \bigl( \mu(C_{n}) \bigr) \bigr) \\ &\leq \psi \bigl(\mu(C_{n}) \bigr), \end{aligned} \end{aligned}$$

for every $n=1,2,\ldots$ . From (1) and condition (A2) of Definition (1.1) we conclude that $\mu(C_{n+1})\leq\mu(C_{n})$ for every $n=1,2,\ldots$ . This means that the sequence $\{\mu(C_{n}) \}_{n=0}^{\infty}$ is not increasing, and consequently there exists $r\geq0$ such that $\lim_{n \to\infty}\mu(C_{n})=r$. Since *ψ* is continuous, according to (2) we get

$$ \psi(r)\leq F \bigl(\psi(r),\phi(r) \bigr)\leq\psi(r), $$

and hence

$$ F \bigl(\psi(r),\varphi(r) \bigr)=\psi(r). $$

The last inequality and the inclusion $F \in\mathcal{C}$ yield $\psi(r)=0$ or $\varphi(r)=0$. These equalities and the inclusions $\psi\in\Psi$, $\varphi\in\Phi$ imply that $r=0$. So, we obtain

3 $$ \lim_{n \to\infty}\mu(C_{n})=0. $$

Let $C_{\infty}=\bigcap_{n=0}^{\infty}C_{n}$. Since $C_{n+1}\subset C_{n}$, $C_{n}$ is bounded, closed, and convex for every $n=0,1, \ldots$ , we see that $C_{\infty}$ is also bounded, closed, and convex, so the equality (3) and property (A5) of Definition (1.1) imply that $C_{\infty}$ is nonempty and compact. From the inclusion (1) it follows that

$$ T(C_{\infty})=T \Biggl(\bigcap_{n=1}^{\infty} C_{n} \Biggr)\subset \Biggl[\bigcap_{n=1}^{ \infty} T(C_{n}) \Biggr]\subset \Biggl[\bigcap_{n=1}^{\infty} C_{n} \Biggr]= C_{\infty}. $$

Finally, by virtue of Schauder’s fixed point theorem we see that the map $T:C_{\infty} \to C_{\infty}$ has a fixed point in $C_{\infty}$. Since $C_{\infty} \subset C$, we conclude that the map *T* has a fixed point in *C*. The proof is completed. □

If we let $F(s,t)=ks$ in Theorem 2.1 we get the following result.

### Corollary 2.2

*Let* *C* *be a nonempty*, *bounded*, *closed*, *and convex subset of a Banach space* *E* *and let* $T : C \to C$ *be a continuous mapping*. *Assume that*

$$ \psi \bigl(\mu( TM) \bigr)\leq k \bigl(\psi \bigl(\mu(M) \bigr) \bigr), $$

*for* $M \subseteq C$. *Then* *T* *has a fixed point*.

If we let $\psi(t)=t$ in Theorem 2.1 we get the following.

### Corollary 2.3

*Let* *C* *be a nonempty*, *bounded*, *closed*, *and convex subset of a Banach space* *E* *and let* $T : C \to C$ *be a continuous mapping*. *Assume that*

$$ \mu( TM)\leq F \bigl(\mu(M),\varphi \bigl(\mu(M) \bigr) \bigr), $$

*for* $M \subseteq C$. *Then* *T* *has a fixed point*.

### Theorem 2.4

*Let* *C* *be a nonempty*, *bounded*, *closed*, *and convex subset of a Banach space* *E* *and let* $T: C \to C$ *be continuous mapping*. *Assume that there exist* $\psi\in\Psi$, $\varphi\in\Phi$ *and* $F \in\mathcal{C}$ *such that the inequality*

$$ \psi \bigl(\mu \bigl(T(C) \bigr) \bigr)\leq F \bigl(\psi \bigl(M(X,Y) \bigr), \varphi \bigl(M(X,Y) \bigr) \bigr), $$

*is satisfied for every noncompact subset* *X*, *Y* *of* *C*, *where*

$$ M(X,Y)=\max \bigl\{ \mu(X),\mu \bigl(T(X) \bigr),\mu \bigl(T(Y) \bigr),\mu \bigl(T(X) \cup T(Y) \bigr) \bigr\} . $$

*Then* *T* *has a fixed point*.

### Proof

Define a sequence $\{C_{n}\}_{n=0}^{\infty}$ setting

$$ C_{0}:=C, \quad\quad C_{n}:=\overline{\operatorname {co}} \bigl(T(C_{n-1}) \bigr), $$

where $n=1,2,\ldots$ . Analogously to Theorem 2.1 it is possible to show that

4 $$ C_{n+1} \subseteq C_{n}, \quad\quad T(C_{n})\subseteq C_{n}, $$

for every $n=0,1,\ldots$ .

If $\mu(C_{N})=0$ for some $N \in\mathbb{N}$ then $C_{N}$ is a compact set and by virtue of Schauder’s theorem the continuous map $T:C_{N} \to C_{N}$ has a fixed point in $C_{N} \subset C$.

Now suppose that $\mu(C_{N})>0$ for every $n \in\mathbb{N}$. From (4) and condition (A2) of Definition 1.1 we conclude that $\mu(C_{n+1})\leq\mu(C_{n})$ for every $n=1,2,\ldots$ . This means that the sequence $\{\mu(C_{n})\}_{n=0}^{\infty}$ is not increasing, and consequently there exists $r\geq0$ such that $\lim_{n \to\infty }\mu(C_{n})=r$.

Taking into consideration that $F \in\mathcal{C}$, condition (A3), we have

5 $$\begin{aligned} \psi \bigl(\mu(C_{n+1}) \bigr) =&\psi \bigl(\mu \bigl( \overline{\operatorname {co}} \bigl(T(C_{n}) \bigr) \bigr) \bigr)= \psi \bigl(T(C_{n}) \bigr) \\ \leq& F \bigl(\psi \bigl(M(C_{n},C_{n+1}) \bigr),\varphi \bigl(M(C_{n},C_{n+1}) \bigr) \bigr) \\ \leq& \psi \bigl(M(C_{n},C_{n+1}) \bigr), \end{aligned}$$

where

$$ M(C_{n},C_{n+1})= \max \bigl\{ \mu(C_{n}),\mu \bigl(T(C_{n}) \bigr),\mu \bigl(T(C_{n+1}) \bigr), \mu \bigl(T(C_{n})\cup T(C_{n+1}) \bigr) \bigr\} . $$

Since $T(C_{n}) \subset C_{n}$ and $C_{n+1}\subset C_{n}$ we have

$$\begin{aligned}& \begin{gathered} \mu \bigl(T(C_{n}) \bigr) \leq\mu(C_{n}), \quad\quad \mu \bigl(T(C_{n+1}) \bigr)\leq\mu \bigl(T(C _{n}) \bigr), \\ \mu \bigl(T(C_{n})\cup T(C_{n+1}) \bigr)\leq \mu(C_{n} \cup C_{n+1})=\mu(C_{n}), \end{gathered} \end{aligned}$$

for every $n\geq1$. Thus we obtain

$$ M(C_{n},C_{n+1})=\mu(C_{n}), $$

and consequently

$$ \psi \bigl(M(C_{n},C_{n+1}) \bigr)=\psi \bigl( \mu(C_{n}) \bigr), \quad\quad \varphi \bigl(M(C_{n},C _{n+1}) \bigr)=\varphi \bigl(\mu(C_{n}) \bigr), $$

for every $n\geq1$. The last equalities and (5) yield

$$ \psi \bigl(\mu(C_{n+1}) \bigr)\leq F \bigl(\psi \bigl(M(C_{n},C_{n+1}) \bigr),\varphi \bigl(M(C_{n},C _{n+1}) \bigr) \bigr)\leq\psi \bigl( \mu(C_{n}) \bigr), $$

and hence

$$ \psi \bigl(\mu(C_{n+1}) \bigr)\leq F \bigl(\psi \bigl( \mu(C_{n}) \bigr),\varphi \bigl(\mu(C_{n}) \bigr) \bigr) \leq \psi \bigl( \mu(C_{n}) \bigr), $$

for every $n\geq1$. Since $\lim_{n \to\infty}\mu(C_{n})=r$; *ψ*, *φ* and *F* are continuous functions, we get

$$ F \bigl(\psi(r),\varphi(r) \bigr)=\psi(r). $$

The inclusion $F \in\mathcal{C}$ yields $\psi(r)=0$ or $\varphi(r)=0$. These equalities and inclusions $\psi\in\Psi$, $\varphi\in\Phi$ imply that $r=0$. So, we obtain

$$ \lim_{n \to\infty}\mu(C_{n})=0. $$

From now on the proof repeats the proof of Theorem 2.1. The theorem is proved. □

By taking $\psi(t)=t$ we have the following.

### Corollary 2.5

*Let* *C* *be a nonempty*, *bounded*, *closed*, *and convex subset of a Banach space* *E* *and let* $T: C \to C$ *be a continuous mapping*. *Assume that there exist* $\psi\in\Psi$, $\varphi\in\Phi$ *and* $F \in \mathcal{C}$ *such that the inequality*

$$ \mu \bigl(T(C) \bigr)\leq F \bigl(\psi \bigl(M(X,Y) \bigr),\varphi \bigl(M(X,Y) \bigr) \bigr), $$

*is satisfied for every noncompact subset* *X*, *Y* *of* *C*, *where*

$$ M(X,Y)=\max \bigl\{ \mu(X),\mu \bigl(T(X) \bigr),\mu \bigl(T(Y) \bigr),\mu \bigl(T(X) \cup T(Y) \bigr) \bigr\} . $$

*Then the map* *T* *has a fixed point*.

### Theorem 2.6

*Let* *C* *be a nonempty*, *bounded*, *closed*, *and convex subset of a Banach space* *E* *and let* $T,S : C \to C$ *be continuous mappings*. *Assume that*:

- *The range of* *T* *contains the range of* *S*.
- *For any* $M \subset C$:
  6 $$ \psi(\mu \bigl( T(M) \bigr)\leq F \bigl(\psi \bigl(\mu \bigl(S(M) \bigr) \bigr),\varphi \bigl(\mu \bigl(S(M) \bigr) \bigr) \bigr), $$

*where* $\psi\in\Psi$, $\varphi\in\Phi$ *and* $F \in\mathcal{C}$.

*Then*:

(i). *The sets* $A=\{x \in C: S(x)=x\}$ *and* $B=\{x \in C: T(x)=x \}$ *are nonempty and closed*.
(ii). *T* *and* *S* *have a coincidence point*.
(iii). *If* *T* *and* *S* *are weakly compatible*. *Then* *S* *and* *T* *have a unique common fixed point*.

### Proof

Let $C_{0}=C$, choose $C_{1}\subset E$ such that $S(C_{0})\subseteq T(C _{1})$ and $C_{1}:=\overline{\operatorname {co}} S(C_{0})$. This can be done since the range of *T* contains the range of *S*. We have

$$ S(C_{0})\subseteq\overline{\operatorname {co}}S(C_{0})\subseteq R_{S}\subseteq R _{T}, $$

so there exists $C_{1}$ such that $S(C_{0})\subseteq T(C_{1})$.

Continuing this process having chosen $C_{n}$ in *E* we obtain $C_{n+1}$ in *E* such that $S(C_{n})\subseteq T(C_{n+1})$ and $C_{n+1}:=\overline{\operatorname {co}} SC_{n}$.

If we put $C_{n+1}:=\overline{\operatorname {co}}S(C_{n})$, then

$$ S(C_{n})\subseteq C_{n+1}=\overline{\operatorname {co}}S(C_{n}) \subseteq C_{0} \quad\textrm{and}\quad T(C_{n+1})\subseteq C_{0},\quad\forall n \in\mathbb{N}\cup\{0\}, $$

so

$$ S(C_{n})\cap T(C_{n+1})\subseteq C_{0}, $$

therefore $S(C_{n})\subseteq T(C_{n+1})$ for every $n\in\mathbb{N} \cup\{0\}$ because the cases

$$ S(C_{n})\cap T(C_{n+1})=\emptyset\quad\textrm{and}\quad T(C_{n+1}) \subseteq S(C_{n}) $$

are impossible, since $R_{S}\subseteq R_{T}$.

We observe that $C_{n+1}\subseteq C_{n}$ and $SC_{n}\subseteq C_{n}$ for $n\in\mathbb{N}\cup\{0\}$, because

$$ C_{1}=\overline{\operatorname {co}} \bigl(S(C_{0}) \bigr) \subseteq C_{0}. $$

Let $C_{n}\subseteq C_{n-1}$ so

$$ C_{n+1}=\overline{\operatorname {co}} \bigl(S(C_{n}) \bigr)\subseteq \overline{\operatorname {co}} \bigl(S(C _{n-1}) \bigr)=C_{n}. $$

And also

$$ S(C_{n})\subset S(C_{n-1})\subset\overline{\operatorname {co}} \bigl(S(C_{n-1}) \bigr)=C _{n}. $$

If $\mu(C_{N})=0$, for some $N \in\mathbb{N}$, then *T* has a fixed point in *C*, because Schauder’s fixed point theorem guarantees this. Suppose $\mu(C_{n})>0$ for each $n \in\mathbb{N}$. Therefore we get

7 $$\begin{aligned} \psi \bigl(\mu(C_{n+1}) \bigr) =&\psi \bigl(\mu \bigl( \overline{\operatorname {co}} (SC_{n}) \bigr) \bigr) \\ =&\psi \bigl(\mu \bigl( \bigl(S(C_{n}) \bigr) \bigr) \bigr) \\ \leq&\psi \bigl(\mu \bigl(T(C_{n+1}) \bigr) \bigr) \\ \leq& F \bigl(\psi \bigl(\mu \bigl(S(C_{n+1}) \bigr) \bigr),\varphi \bigl( \mu \bigl(S(C_{n+1}) \bigr) \bigr) \bigr) \\ \leq& F \bigl(\psi \bigl(\mu(C_{n+1}) \bigr),\varphi \bigl( \mu(C_{n+1}) \bigr) \bigr) \\ \leq& \psi \bigl(\mu(C_{n+1}) \bigr). \end{aligned}$$

Since $C_{n+1}\subset C_{n}$ for every $n=1,2,\ldots$ , the condition (A2) of Definition 1.1 implies that $\mu(C_{n+1})\leq\mu(C _{n})$ for every $n=1,2,\ldots$ . This means that the sequence $\{\mu(C_{n})\}_{n=0}^{\infty}$ is not increasing, and consequently there exists $r\geq0$ such that $\lim_{n \to\infty}\mu(C_{n})=r$. By (7) we find that

$$ \psi(r)\leq F \bigl(\psi(r),\varphi(r) \bigr)\leq\psi(r), $$

so

$$ F \bigl(\psi(r),\varphi(r) \bigr)=\psi(r), $$

according to the property of *F* we have $\psi(r)=0$ or $\varphi(r)=0$. Hence

$$ r=\lim_{n \to\infty} \mu(C_{n})=0. $$

Also since $C_{n+1}\subseteq C_{n}$ by property (A5) of Definition 1.1 $C_{\infty}=\bigcap_{n=1}^{\infty}C_{n}$ is nonempty and compact.

Moreover, since $C_{n}$ and *C* are convex, and $S(C_{n})\subset C _{n}$, $S:C_{n} \to C_{n}$ for $n=0,1,2,\ldots$ and so $S:C_{\infty } \to C_{\infty}$, now Schauder’s fixed point theorem ensures *S* has a fixed point and the set $A=\{x \in C: S(x)=x\}$ is nonempty and closed.

Similarly to *S*; *T* has a fixed point and by continuity of *T*, $B=\{x \in C: T(x)=x\}$ is nonempty and closed. By Lemma 1.7, take

$$ D:= \bigl\{ x \in C: S(x)=x \textrm{ or } T(x)=x \bigr\} $$

and define a map $g:=S(D) \to S(D)$ by $g(Sx)=Tx$. Clearly *g* is well defined. Now if we put $X:S(D)$ and $E:=B$ in Lemma 1.7, then $g(E)=g(X)$, so *g* is one to one. Now by using (6) we have

$$ \psi \bigl(\mu \bigl(g \bigl(S(M) \bigr) \bigr) \bigr)=\psi \bigl(\mu \bigl(T(M) \bigr) \bigr)\leq F \bigl(\psi \bigl(\mu \bigl(S(M) \bigr) \bigr),\varphi \bigl( \mu \bigl(S(M) \bigr) \bigr) \bigr), $$

so according to Theorem 2.1 there exists $z \in E$ and it is unique, since *g* is one to one, such that $g(Sz)=Sz$, which implies $Tz=Sz$.

Hence *T* and *S* have a unique coincidence point thus from Proposition 1.6 and it follows that *T* and *S* have a unique common fixed point. □

### Example 2.7

Let $C=[0,2]$ be a subset of $\mathbb{R}$. Take $S,T:[0,2] \to[0,2]$ defined by

$$ T(x)=\frac{1}{2}x \quad\mbox{and} \quad S(x)=\frac{1}{3}x, $$

also let

$$ M=[1,2], \quad\quad \varphi(t)=\frac{t}{6}, \quad\quad \psi(t)=t, \quad\quad F(s,t)= \frac{s}{s+t}, $$

and

$$ \mu(X)=\operatorname {diam}(X). $$

It is clear that $\psi\in\Psi$ and $\varphi\in\Phi$. To verify the hypotheses of Theorem 2.6:

- $\mathbf{R}_{S}=[0, \frac{2}{3}] \subset\mathbf {R}_{T}=[0,1]$.
  By taking $C_{0}=[0,2]$ and $C_{1}=[0,\frac{4}{3}]$ we have
  $$ S \bigl([0,2] \bigr)= \biggl[0,\frac{2}{3} \biggr]=T \biggl( \biggl[0, \frac{4}{3} \biggr] \biggr), $$
  the algorithm of $C_{n}$ follows by
  $$ C_{3}= \biggl[0,\frac{16}{27} \biggr],\quad\quad C_{4}= \biggl[0,\frac{32}{81} \biggr],\quad\quad C_{5}= \biggl[0, \frac{64}{243} \biggr],\quad\quad \cdots, $$
  such that $S(C_{n})\subseteq T(C_{n+1})$.
- $\psi(\mu(T[1,2]))=\frac{1}{2}$, $\psi(\mu(S[1,2]))= \frac{1}{3}$, $\varphi(\mu(S[1,2]))=\frac{1}{18}$, and also
  $$ F \biggl(\frac{1}{3},\frac{1}{18} \biggr)=\frac{6}{7}, $$
  therefore
  8 $$ \psi \biggl(\mu \bigl( T(M) \bigr)=\frac{1}{2}\leq F \bigl(\psi \bigl(\mu \bigl(S(M) \bigr) \bigr),\varphi \bigl(\mu \bigl(S(M) \bigr) \bigr) \bigr) \biggr)= \frac{6}{7}, $$

thus the sets $F=\{x \in C: S(x)=x\}$ and $K=\{x \in C: T(x)=x\}$ are nonempty and closed. Also $T(0)=S(0)$, so 0 is a coincidence point. Finally since *T* and *S* commute at 0, that is, $ST(0)=TS(0)$, so *T* and *S* are weakly compatible and 0 is a common fixed point of *S* and *T*.

### Theorem 2.8

*Let* *C* *be a nonempty*, *bounded*, *closed*, *and convex subset of a Banach space* *E* *and let* $T: C \to C$ *be a continuous mapping such that*

9 $$ \int_{0}^{\varphi(\mu(T(M)))}f(t)\,dt\leq F \biggl( \int_{0}^{\varphi( \mu(M))}f(t)\,dt, \int_{0}^{\psi(\mu(M))}f(t) \biggr) , $$

*for any subset* *M* *of* *C* *and where* $f:[0, \infty) \to[0, \infty)$ *be a Lebesgue integrable function*, *which is summable on each compact of* $[0, \infty)$ *and* $\int_{0}^{\varepsilon} f(t)\,dt >0$ *for each* $\varepsilon> 0$ *and* $\psi\in\Psi$, $\varphi\in\Phi$ *and* $F \in\mathcal{C}$. *Then* *T* *has a fixed point*.

### Proof

Define a sequence $\{C_{n}\}$ as follows:

$$ C_{0}:=C, \quad\quad C_{n}:=\overline{\operatorname {co}} TC_{n-1}, \quad\mbox{for } n=1,2,\ldots. $$

If $\mu(C_{N})=0$ for some $N \in\mathbb{N}$. Then *T* has a fixed point *C* by the proof of previous theorems. Suppose $\mu(C_{n})>0$ for all $n \in\mathbb{N}$. Since $C_{n+1}\subset C_{n}$ for every $n=1,2,\ldots$ , the condition (A2) of Definition 1.1 implies that $\mu(C_{n+1})\leq\mu(C_{n})$ $n=1,2,\ldots$ . This means that the sequence $\{\mu(C_{n})\}_{n=0}^{\infty}$ is not increasing, and consequently there exists $r\geq0$ such that $\lim_{n \to\infty} \mu(C_{n})=r$. From the inclusions $\psi\in\Psi$, $\varphi\in \Phi$ we obtain $\lim_{n \to\infty}\psi(\mu(C_{n}))=\psi(r)$ and $\lim_{n \to\infty}\varphi(\mu(C_{n}))=\varphi(r)$. Since $f(\cdot)$ is Lebesgue integrable on each compact subset of $[0,\infty)$, we see that

$$ \lim_{n \to\infty} \int_{0}^{\psi(\mu(C_{n}))}f(t)\,dt= \int_{0}^{ \psi(r)}f(t)\,dt, \quad\quad \lim _{n \to\infty} \int_{0}^{\varphi(\mu(C _{n}))}f(t)\,dt= \int_{0}^{\varphi(r)}f(t)\,dt. $$

The last equalities, inclusion $F \in\mathcal{C}$ and (9) imply that

$$ \int_{0}^{\varphi(r)}f(t)\,dt \leq F \biggl( \int_{0}^{\varphi(r)}f(t)\,dt, \int_{0}^{\psi(r)}f(t)\,dt \biggr)\leq \int_{0}^{\varphi(r)}f(t)\,dt, $$

and hence

$$ F \biggl( \int_{0}^{\varphi(r)}f(t)\,dt, \int_{0}^{\psi(r)}f(t)\,dt \biggr)= \int_{0}^{\varphi(r)}f(t)\,dt. $$

Thus we see that

$$ \int_{0}^{\varphi(r)}f(t)\,dt=0, \quad\mbox{or} \quad \int_{0}^{ \psi(r)}f(t)\,dt=0, $$

and consequently

$$ \varphi(r)=0 \quad \text{or} \quad\psi(r)=0. $$

From the last equalities it follows that $r=0$, *i.e.* $\lim_{n \to\infty}\mu(C_{n})=r$. Also since $C_{n+1}\subseteq C _{n}$ by property (A5) of Definition 1.1 $C_{\infty}=\bigcap_{n=1}^{\infty}C_{n}$ is a nonempty, closed, and convex subset of *C*. Moreover, we know that $C_{\infty}$ belongs to $\ker(\mu)$. So $C_{\infty}$ is compact and invariant by the mapping *T*. Consequently, Schauder’s fixed point theorem implies that *T* has a fixed point in $C_{\infty}$. Since $C_{\infty}\subset C$ the proof is complete. □

### Remark 2.9

Put $f(t)=1$, $\varphi(t)=t$ and $F(s,t)=ks$ for $t \in[0,\infty)$ in Theorem 2.8. Then

$$\begin{aligned} \mu \bigl(T(X) \bigr) =& \int_{0}^{\varphi(\mu(T(X)))}f(t)\,dt \\ \leq& F \biggl( \int_{0}^{\varphi(\mu(X))}f(t)\,dt, \int_{0}^{\psi(\mu(X))}f(t)\,dt \biggr) \\ = &k \biggl( \int_{0}^{\varphi(\mu(X))}f(t)\,dt \biggr) \\ =&k \bigl(\mu(X) \bigr), \end{aligned}$$

thus we get Darbo’s fixed point theorem.

### Corollary 2.10

*Let* *C* *be a nonempty*, *bounded*, *closed*, *and convex subset of a Banach space* *E* *and let* $T: C \to C$ *be a continuous mapping such that*

$$ \int_{0}^{\varphi(\Vert Tx-Ty\Vert )}f(t)\,dt\leq F \biggl( \int_{0}^{\varphi(\Vert x-y\Vert )}f(t)\,dt, \int_{0}^{\psi(\Vert x-y\Vert )}f(t)\,dt \biggr), $$

*for any* $x,y \in C$ *where* $f:[0, \infty) \to[0, \infty)$ *be a Lebesgue integrable function which is summable on each compact set of* $[0, \infty)$ *and* $\int_{0}^{\varepsilon} f(t)\,dt > 0$ *for each* $\varepsilon> 0$ *and* $\psi\in\Psi$, $\varphi\in\Phi$ *and* $F \in\mathcal{C}$. *Then* *T* *has a fixed point*.

### Proof

Define $\mu:\mathfrak{m}_{E} \to\mathbb{R}_{+}$ with $\mu(X)=\operatorname {diam}(X)$ for any $X \in\mathfrak{m}_{E}$, where $\operatorname {diam}(X)$ is a diameter of the set *X*. It is easy to verify that *μ* is a measure of noncompactness on space *E*. By assumption, we have

$$ \int_{0}^{\varphi(\sup_{x,y \in X}\Vert Tx-Ty\Vert )}f(t)\,dt \leq F \biggl( \int_{0} ^{\varphi(\sup_{x,y \in X}\Vert x-y\Vert )}f(t)\,dt, \int_{0}^{\psi( \sup_{x,y \in X}\Vert x-y\Vert )}f(t)\,dt \biggr), $$

thus we get

$$ \int_{0}^{\varphi(\mu(TX))}f(t)\,dt\leq F \biggl( \int_{0}^{\varphi(\mu(X))}f(t)\,dt, \int_{0}^{\psi(\mu(X))}f(t)\,dt \biggr), $$

so according to Theorem 2.8 we get the result. □

### Corollary 2.11

*Let* *C* *be a nonempty*, *bounded*, *closed*, *and convex subset of a Banach space* *E* *and let* $T: C \to C$ *be continuous mapping such that*

$$ \int_{0}^{\varphi(\mu(T(X)))}f(t)\,dt\leq F \biggl( \int_{0}^{\varphi(\mu(X))}f(t)\,dt, \int_{0}^{\psi(\mu(X))}f(t)\,dt \biggr)-\psi^{*} \biggl( \int_{0}^{\psi(\mu(X))}f(t)\,dt \biggr), $$

*for any subset* *X* *of* *C* *and where* $f:[0, \infty) \to[0, \infty)$ *be a Lebesgue integrable function*, *which is summable on each compact subset of* $[0, \infty)$ *and* $\int_{0}^{\varepsilon} f(t)\,dt>0$ *for each* $\varepsilon> 0$ *and* $\psi,\psi^{*} \in\Psi$, $\varphi\in\Phi$ *and* $F \in\mathcal{C}$. *Then* *T* *has a fixed point*.

### Proof

Define a sequence $\{C_{n}\}_{n=0}^{\infty}$ setting

$$ C_{0}:=C, \quad\quad C_{n}:=\overline{\operatorname {co}} T(C_{n-1}), $$

where $n=1,2,\ldots$ . Analogously to Theorem 2.1 it is possible to show that

10 $$ C_{n+1} \subseteq C_{n}, \quad\quad T(C_{n})\subseteq C_{n}, $$

for every $n=0,1,\ldots$ . From condition (A2) of Definition 1.1 we conclude that $\mu(C_{n+1})\leq\mu(C_{n})$ for every $n=1,2, \ldots$ . This means that the sequence $\{\mu(C_{n})\}_{n=0}^{\infty }$ is not increasing, and consequently there exists $r\geq0$ such that $\lim_{n \to\infty}\mu(C_{n})=r$.

If $\mu(C_{N})=0$ for some $N \in\mathbb{N}$ then $C_{N}$ is a compact set and using Schauder’s theorem we conclude that the continuous map $T:C_{N} \to C_{N}$ has a fixed point in $C_{N} \subset C$.

Now suppose that $\mu(C_{N})>0$ for every $n \in\mathbb{N}$. Taking into consideration that $F \in\mathcal{C}$, $\psi^{*} \in\psi$ and condition (A3), we have

11 $$\begin{aligned} \int_{0}^{\varphi(\mu(C_{n+1}))}f(t)\,dt =& \int_{0}^{\varphi(\mu(\overline{ \operatorname {co}}(TC_{n})))}f(t)\,dt \\ =& \int_{0}^{\varphi(\mu(TC_{n}))}f(t)\,dt \\ \leq&F \biggl( \int_{0}^{\varphi(\mu(C_{n}))}f(t)\,dt, \int_{0}^{\psi( \mu(C_{n}))}f(t)\,dt \biggr)-\psi^{*} \biggl( \int_{0}^{\psi(\mu(C_{n}))}f(t)\,dt \biggr) \\ \leq& \int_{0}^{\varphi(\mu(C_{n}))}f(t)\,dt-\psi^{*} \biggl( \int_{0} ^{\psi(\mu(C_{n}))}f(t)\,dt \biggr) . \end{aligned}$$

Since $\lim_{n \to\infty}\mu(C_{n})=r$, $F \in\mathcal{C}$, $\varphi\in\Phi$, $\psi^{*} \in\Psi$, the function $f(\cdot)$ is Lebesgue summable on the compact subset of $[0,\infty)$, and from (11) we obtain

$$ \int_{0}^{\varphi(r)}f(t)\,dt\leq \int_{0}^{\varphi(r)}f(t)\,dt - \psi ^{*} \biggl( \int_{0}^{\psi(r)}f(t)\,dt \biggr) , $$

and hence

$$ \psi^{*} \biggl( \int_{0}^{\psi(r)}f(t)\,dt \biggr) \leq0. $$

From the last inequality it follows that $\psi(r)=0$ and consequently $r=0$, *i.e.* $\lim_{n \to\infty}\mu(C_{n})=0$.

Continuing the proof analogously to the proof of the theorem 2.1 we obtain the result. □

### Theorem 2.12

*Let* *C* *be a nonempty*, *bounded*, *closed*, *and convex subset of a Banach space* *E* *and let* $T,S : C \to C$ *be continuous mappings*. *Assume that*:

- *The range of* *T* *contains the range of* *S*.
- *For any* $M \subseteq C$:
  12 $$ \int_{0}^{\varphi(\mu( T(M))}f(t)\,dt \leq F \biggl( \int_{0}^{\varphi( \mu(S(M)))}f(t)\,dt, \int_{0}^{\psi(\mu(S(M)))}f(t)\,dt \biggr), $$

*where* $\psi\in\Psi$, $\varphi\in\Phi$ *and* $F \in\mathcal{C}$.

*Then*:

(i). *The sets* $A=\{x \in C: S(x)=x\}$ *and* $B=\{x \in C: T(x)=x \}$ *are nonempty and closed*.
(ii). *T* *and* *S* *have a coincidence point*.
(iii). *If* *T* *and* *S* *are weakly compatible*, *then* *S* *and* *T* *have a common fixed point*.

### Proof

Let $C_{0}=C$, $C_{n}:=\overline{\operatorname {co}} SC_{n-1}$ for $n=1,2,\ldots$ . As is pointed out in Theorem 2.6, $C_{n+1}$ in *E* such that $T(C_{n+1})=S(C_{n})$. We have

$$ T(C_{n})\subseteq C_{n}\quad\text{and also}\quad S(C_{n})\subset S(C _{n-1})\subset\overline{\operatorname {co}} \bigl(S(C_{n-1}) \bigr)=C_{n}, $$

for all $n\in\mathbb{N}$. Thus

13 $$\begin{aligned} \int_{0}^{\varphi(\mu(C_{n+1}))}f(t)\,dt =& \int_{0}^{\varphi(\mu(\overline{ \operatorname {co}} (S(C_{n}))))}f(t)\,dt \\ =& \int_{0}^{\varphi(\mu(S(C_{n})))}f(t)\,dt \\ =& \int_{0}^{\varphi(\mu(T(C_{n+1})))}f(t)\,dt \\ \leq& F \biggl( \int_{0}^{\varphi(\mu(S(C_{n+1})))}f(t)\,dt, \int_{0}^{ \psi(\mu(S(C_{n+1})))}f(t)\,dt \biggr) \\ \leq& F \biggl( \int_{0}^{\varphi(\mu(C_{n+1}))}f(t)\,dt, \int_{0}^{\psi( \mu(C_{n+1}))}f(t)\,dt \biggr) \\ \leq& \int_{0}^{\varphi(\mu(C_{n+1}))}f(t)\,dt \\ \leq& \int_{0}^{\varphi(\mu(C_{n}))}f(t)\,dt. \end{aligned}$$

The remaining part is similar to the previous proofs. □

## Application

In this section, since integral equations arise in different problems of theory and applications (see, *e.g.*, [19–21] and the references therein). We consider the existence of solutions for the following nonlinear integral equation in $\operatorname {BC}(\mathbb{R}^{+})$, the space of bounded and continuous functions $x(\cdot): \mathbb{R}^{+} \to \mathbb{R}^{+}$:

14 $$ x(t)=\lambda f \biggl(t, \int_{0}^{t}g \bigl(t,s,x \bigl(\alpha(s) \bigr) \bigr) \,ds \biggr)+(1-\lambda) \int _{0}^{t}g \bigl(t,s,x(s) \bigr)\,ds, \quad t \geq0, \lambda\in(0,1) $$

for any nonempty bounded subset *X* of $\operatorname {BC}(\mathbb{R}^{+})$, $x \in X$ and $T>0$ and $\epsilon>0$, let

$$\begin{aligned}& \omega^{T}(x,\epsilon)=\sup \bigl\{ \bigl\vert x(t)-x(s) \bigr\vert : s,t \in[0,T], \vert t-s\vert \leq\epsilon \bigr\} , \\& \omega^{T}(X,\epsilon)=\sup \bigl\{ \omega^{T}(x,\epsilon): x \in X \bigr\} , \quad\quad \omega_{0}^{T}(X)=\lim _{\epsilon\to0}\omega^{T}(X,\epsilon), \\& \omega_{0}(X)=\lim_{T \to\infty}\omega_{0}^{T}(X), \quad\quad X(t)= \bigl\{ x(t): x \in X \bigr\} , \\& \operatorname {diam}X(t)=\sup \bigl\{ \bigl\vert x(t)-y(t) \bigr\vert : x,y \in X \bigr\} , \end{aligned}$$

and

15 $$ \mu(X)=\omega_{0}(X)+ \lim_{t \to\infty}\sup \operatorname {diam}X(t). $$

Banaś has shown in [13] that the function *μ* is a measure of noncompactness in the space $\operatorname {BC}(\mathbb{R}^{+})$.

### Theorem 3.1

*The nonlinear integral equation* (14) *has at least one solution in the space* $\operatorname {BC}(\mathbb{R}^{+})$, *if the following conditions are satisfied*:

$(A_{0})$.
*The function* $\alpha:\mathbb{R}^{+} \to\mathbb{R} ^{+}$ *is continuous*, $\alpha(t) \to\infty$ *as* $t \to\infty$.
$(A_{1})$.
*The function* $f:\mathbb{R}^{+} \times\mathbb{R} \to\mathbb{R}$ *is continuous and*
  $$ \bigl\vert f(t,x)-f(t,y) \bigr\vert \leq\psi \bigl(\vert x-y\vert \bigr), $$
  *moreover*, *ψ* *and* *φ* *are an altering distance function and an ultra altering distance function*, *respectively which* *ψ* *satisfies for all* $t,s \in\mathbb{R}^{+}$, $\psi(t)+\psi(s)\leq \psi(s+t)$ *and* $\psi(t)< t$.
$(A_{2})$.
  $$ L=\sup \bigl\{ f(t,0): t \in\mathbb{R}^{+} \bigr\} < \infty. $$
$(A_{3})$.
*The function* $g:\mathbb{R}^{+} \times\mathbb{R} ^{+} \times\mathbb{R} \to\mathbb{R}$ *is continuous and there exists a continuous function* $b:\mathbb{R}^{+} \times\mathbb{R}^{+} \to \mathbb{R}^{+}$ *which is increasing with respect to the first component*, *satisfying*
  $$ \bigl\vert g(t,s,x) \bigr\vert \leq b(t,s), $$
  *for all* $t,s \in\mathbb{R}^{+}$ *and* $x \in\mathbb{R}$ *where*
  $$ \lim_{t \to\infty} \int_{0}^{t} b(t,s)\,ds=0. $$
$(A_{4})$.
  $$ f \bigl(t,Kx(t) \bigr)=K \bigl(f \bigl(t,x(t) \bigr) \bigr), $$
  *where*
  $$ Kx(t)= \int_{0}^{t} g \bigl(t,s,x(s) \bigr)\,ds. $$

For the following remark, by definition, commuting mappings means for a pair of self-mappings $K,L:X \to X$ that there exists a point $x \in X$ such that $KL(x)=LK(x)$.

### Remark 3.2

Note that by hypothesis $(A_{3})$ there exists constant $V >0$ such that

$$ V=\sup_{t\geq0} v(t)=\sup_{t \geq0} \biggl[ \int_{0}^{t} b(t,s)\,ds \biggr]. $$

### Proof

Put

$$\begin{aligned}& Q= \bigl\{ x \in \operatorname {BC}\bigl(\mathbb{R}^{+} \bigr): \Vert x\Vert \leq r= L+V \bigr\} , \\& (Fx) (t)=f \bigl(t,x(t) \bigr),\quad\quad (Kx) (t)= \int_{0}^{t} g \bigl(t,s,x(s) \bigr)\,ds. \end{aligned}$$

Thus equation (14) becomes

$$ x(t)=(Hx) (t)=\lambda FKx \bigl(\alpha(t) \bigr)+(1-\lambda) (Kx) (t) $$

we define the operation $G:C(\mathbb{R}^{+}) \to C(\mathbb{R}^{+})$ by

$$ G(x)=\frac{Hx-(1-\lambda)(Kx)(t)}{\lambda}=FKx \bigl(\alpha(t) \bigr), $$

where $C(\mathbb{R}^{+})$ is the space of continuous functions on $\mathbb{R}^{+}$.

Khodabakhshi and Vaezpour in [6] have shown that *G*, *K* are continuous on *Q*, bounded, commuting mappings, $R_{G}\subseteq R_{K}$ and also *G*, *K* have a common fixed point. The conditions (a) and (b) of Theorem 2.6 hold.

By referring to [6] we get

$$ \mu \bigl(G(X) \bigr) \leq\psi \bigl(\mu \bigl(K(X) \bigr) \bigr)\leq\mu \bigl(K(X) \bigr). $$

On the other hand

$$ \bigl\vert g(t,s,x) \bigr\vert \leq b(t,s), $$

for all $t,s \in\mathbb{R}^{+}$. And for $T>0$ such that $t_{1},t _{2}\in[0,T]$ and $x\in X$ we have

16 $$\begin{aligned} \bigl\vert Kx(t_{2})-Kx(t_{1}) \bigr\vert =& \biggl\vert \int_{0}^{t_{2}}g \bigl(t_{2},s,x(s) \bigr) \,ds- \int _{0}^{t_{1}}g \bigl(t_{1},s,x(s) \bigr) \,ds \biggr\vert \\ \leq& \biggl\vert \int_{0}^{t_{2}}g \bigl(t_{2},s,x(s) \bigr) \,ds \biggr\vert \\ \leq& \int_{0}^{t_{2}}b(t_{2},s)\,ds \\ \leq& \int_{0}^{T}b(T,s)\,ds, \end{aligned}$$

so

17 $$ \omega_{0}^{T} (KX)\leq \int_{0}^{T} b(T,s)\,ds; $$

by taking $T \to\infty$

18 $$ \omega_{0}(KX)\leq\lim_{T \to\infty} \int_{0}^{T} b(T,s)\,ds=0. $$

Also

19 $$\begin{aligned} \bigl\vert Kx(t)-Ky(t) \bigr\vert =& \biggl\vert \int_{0}^{t}g \bigl(t,s,x(s) \bigr)\,ds- \int_{0}^{t}g \bigl(t,s,y(s) \bigr)\,ds \biggr\vert \\ \leq& \biggl\vert \int_{0}^{t}g \bigl(t,s,x(s) \bigr)\,ds \biggr\vert \\ \leq& \int_{0}^{t}b(t,s)\,ds \\ \leq& \int_{0}^{T}b(T,s)\,ds, \end{aligned}$$

so we have

20 $$ \limsup_{T \to\infty} \operatorname {diam}\bigl(KX(T) \bigr) \leq \limsup_{T \to\infty} \int _{0}^{T} b(T,s)\,ds=0; $$

by (18) and (20) we have

$$ \mu(KX)=\mu \Bigl(\omega_{0}(KX)+\limsup_{T \to\infty} \operatorname {diam}(KX) \Bigr)= \mu(0)+0=0, $$

therefore

$$ \psi \bigl(\mu(KX) \bigr)=0, $$

thus for the $\mathcal{C}$-class function *F* and the ultra altering distance function *φ*, defined in Definitions 1.4 and 1.3, respectively, we can get

$$ \psi \bigl(\mu \bigl(G(X) \bigr)\leq\psi \bigl(\mu \bigl(K(X) \bigr) \bigr)=F \bigl(\psi \bigl(\mu(KX) \bigr),\varphi \bigl( \mu(KX) \bigr) \bigr) \bigr), $$

since *K* and *G* commutes, so they are weakly compatible; therefore according to Theorem 2.6, *G* and *K* have a common fixed point so *H* has a fixed point and thus the functional integral equation (14) has at least one solution. □

## Conclusion

In Theorem 3.2 of [6] the condition $TS=ST$ for two self-mappings *T*, *S* is used. But in this article for achieving a common fixed point from two self-maps the hypothesis of the common range which is weaker than the hypotheses of a commuting map is utilized.

In this article the measure of noncompactness is used instead of the metric *d*, which is used in [4] and [11]. Also, contractions associated with a measure of noncompactness in two linear and integral types and the application in solving integral equations are considered, while in the two mentioned article just the linear contractions in terms of the metric *d* is used for achieving a fixed point.
